# Supplementary material for: Tailoring Digital Tools to Address the Radiation and Health Information Needs of Returnees after a Nuclear Accident
Source: Int J Environ Res Public Health. 2021 Dec 2;18(23):12704. doi: 10.3390/ijerph182312704 (PMC8656648; doi:10.3390/ijerph182312704)
Supplement: Supplementary file 1 [file ijerph-18-12704-s001.zip › ijerph-1452255-supplementary.pdf]

Table S1. Recommendations of the SHAMISEN-SINGS project on the design of applications for health and well-being in a nuclear accident [5].

No.	Recommendations
1	Optimize content with stakeholders
2	Balance content, security, and development cost
3	Develop a user support system
4	Apply incentives to promote usage
5	Respond to queries about radiation and health
6	Involve vulnerable populations
7	Accommodate multiple languages
8	Consider ethical issues, especially privacy protection
